# A timeline of cognitive functioning in glioma patients who undergo awake brain tumor surgery

**DOI:** 10.1007/s00701-023-05588-5

**Published:** 2023-04-25

**Authors:** A.M. de Sain, M.H.M. Mantione, I.M.C. Huenges Wajer, M.J.E. van Zandvoort, P.W.A. Willems, P.A. Robe, C. Ruis

**Affiliations:** 1 Department of Experimental Psychology, Utrecht University, Heidelberglaan 1, 3584 CS Utrecht, the Netherlands; 2 Department of Neurology & Neurosurgery, University Medical Center Utrecht, Heidelberglaan 100, 3584 CX Utrecht, the Netherlands

**Keywords:** Craniotomy, Cognition, Brain tumor, Glioma

## Abstract

**Background:**

The purpose of awake brain tumor surgery is to maximize the resection of the tumor and to minimize the risk of neurological and cognitive impairments. The aim of this study is to gain understanding of the development of possible postoperative cognitive deficits after awake brain tumor surgery in patients with suspected gliomas, by comparing preoperative, early postoperative, and late postoperative functioning. A more detailed timeline will be helpful in informing candidates for surgery about what to expect regarding their cognitive functioning.

**Methods:**

Thirty-seven patients were included in this study. Cognitive functioning was measured by means of a broad cognitive screener preoperatively, days after surgery and months after surgery in patients who underwent awake brain tumor surgery with cognitive monitoring. The cognitive screener included tests for object naming, reading, attention span, working memory, inhibition, inhibition/switching, and visuoperception. We performed a Friedman ANOVA to analyze on group level.

**Results:**

Overall, no significant differences were found between preoperative cognitive functioning, early postoperative cognitive functioning, and late postoperative cognitive functioning, except for performances on the inhibition task. Directly after surgery, patients were significantly slower on this task. However, in the following months after surgery, they returned to their preoperative level.

**Conclusion:**

The timeline of cognitive functioning after awake tumor surgery appeared overall stable in the early and late postoperative phase, except for inhibition, which is more difficult in the first days after awake brain tumor surgery. This more detailed timeline of cognitive functioning, in combination with future research, can possibly be contributing in informing patients and caregivers what to expect after awake brain tumor surgery.

## Introduction

Awake brain tumor surgery is a safe and feasible procedure to prevent neurological deficits [8, 9, 14]. The purpose of brain tumor surgery is to maximize the resection of the tumor while minimizing the risk of postoperative cognitive impairments. In awake brain tumor surgery, this is achieved through intraoperative cortical and subcortical electrostimulation in an awake patient [21, 35]. Awake brain surgery is especially useful when the tumor is located in an eloquent area [10, 31]. Awake surgery is associated with a shorter hospital stay, fewer neurological deficits, and shorter surgery time compared to surgery under general anesthesia [3].

Several studies investigated cognitive functioning after awake brain surgery [5, 22, 23, 30] and did not find evident postoperative deterioration in neurocognitive functioning after an awake procedure [22, 30]. For instance, the study of Van Kessel et al. [30] showed preserved cognitive functioning on most neurocognitive domains (executive functioning, memory, language, and visuospatial functioning), except for psychomotor speed. However, most studies only assessed pre- versus postoperative neuropsychological functioning, administrated months after surgery [22, 30]. A more detailed timeline of cognitive functioning is currently missing, particularly when it comes to the immediate postoperative phase. Due to fatigue or swelling of the brain in the first days after surgery, cognitive functioning may (temporarily) deteriorate. It is, however, unclear whether this is the case and if so, how and in what pace this deterioration will recover. More information about the course of cognitive functioning after awake brain tumor surgery can be used in psychoeducation, which contributes to patient’s cognitive, physical, and psychological wellbeing [18].

The aim of this prospective study is to provide more insight in the course of cognitive functioning in the first days and months after awake brain tumor surgery in glioma patients in comparison to the preoperative level. Cognitive functioning was measured by a concise cognitive screener that taps the cognitive functions object naming, reading, attention span, working memory, inhibition, inhibition/switching, and visuoperception. The cognitive screener was performed at three moments in time: days before surgery, days after surgery, and months after surgery. To elaborate the timeline even more, an additional measurement during surgery was taken into account if that proves feasible. Feasibility of this last mentioned measurement depended on mental and physical condition, planning and logistic reasons. The cognitive performances on the different moments were compared to each other.

## Methods

### Participants

In total, 49 patients with suspected diffuse glioma were candidate for awake brain tumor surgery between January 2021 and December 2021 at our center. All patients gave written informed consent. The data of 12 patients could not be used in the final analysis because of missing data (see Appendix A for reasons missing data). Table 1 summarizes the demographic and clinical characteristics of the patients (*n* = 37) that were included in the analyses. Patients (57% male) had an average age of 53 years (SD = 13), the youngest patient was 24 years old and the oldest 79. Thirty-eight percent of the patients had a lower level of education and 62% had a higher level of education. Most of the patients were right-handed (78%), followed by left-handed (14%) and ambidexterity (8%). Twenty patients had a World Health Organization (WHO) grade IV tumor. Other patients had a WHO grade tumor II and III. The location of brain tumors between the left and right hemispheres was approximately equal. Tumors were mostly in the frontal lobe, followed by the parietal, temporal and occipital lobe. Some tumors were not restricted to one lobe, and their locations were defined as temporal/insular and frontal/temporal/insular.

**Table 1:** Demographic and clinical characteristics of the patients included in the analyses (*n* = 37)

Male (%)	21 (56.8)
Age in years; mean ± SD, range	53± 13, 24–79
Educational level^a^	
Low (1–5) (%)	14 (37.9)
High (6–7) (%)	23 (62.1)
Mean ± SD (range)	5.65 ± 1.14 (3–7)
Median (IQR 25–75)	6 (5-6.5)
Handedness	
Right (%)	29 (78.4)
Left (%)	5 (13.5)
Ambidexterity (%)	3 (8.1)
WHO grade II (%)	11 (29.7)
WHO grade III (%)	6 (16.2)
WHO grade IV (%)	20 (54.1)
Tumor left hemisphere (%)	20 (54)
Tumor right hemisphere (%)	17 (46)
Tumor location	
Frontal (%)	14 (37.8)
Parietal (%)	10 (27.0)
Temporal (%)	9 (24.3)
Occipital (%)	1 (2.7)
Temporal/insular (%)	2 (5.4)
Frontal/temporal/insular (%)	1 (2.7)
Male (%)	21 (56.8)

^a^Educational level according to the Verhage Classification [32]

*IQR* Interquartile range; *SD* standard deviation

Inclusion criteria were a good command of the Dutch language and an age over 18 years. Also, only patients who were able to complete at least two out of four measurements were included in this study. Patients received an information letter about this study a few days before the first appointment with the neuropsychologist. All patients were asked to provide informed consent prior to the study to allow the use of their anonymized data. The data collection for this study did not request any additional procedure for the patient, because the screener was already part of clinical care as usual. This study was presented to the Medical Research Ethics Committee of the University Medical Center Utrecht, who concluded that the Medical Research Involving Human Subjects Act (WMO) did not apply to this study and therefore an official approval by the Medical Research Ethics Committee UMC Utrecht was not required.

### Materials

A cognitive screener was compiled of existing neuropsychological tasks, measuring object naming, reading, attention span, working memory, inhibition, inhibition/switching, and visuoperception. Four parallel versions were available with similar levels of difficulty [19].

Object naming was assessed by using 15 pictures from the Snodgrass Naming Task [28]. The numbers of correctly named objects were gathered. The reading task consisted of reading five words, five sentences, and a short story. The numbers of correctly read words and sentences were scored, just as the number of reading errors in the short story. Next, items based on the Digit Span Backward and Forward (span 3-4-5) [34] were used to measure attention span and working memory. During the forward condition (attention span task), patients were asked to repeat digits in the same order. The sequence of the digits started with three and increased to four and five. In the backward condition (working memory task), patients were asked to repeat the digits in the reverse order. The span started with two and increased to three and four digits. The highest score out of three from both the backward and forward condition were scored. In addition, 2 × 12 items based on the Delis-Kaplan Executive Function System (DKEFS) Colour-Word Interference Test (CWIT) were used [11] to measure inhibition and switching. In the first condition (inhibition), patients were asked to name the color of the ink and not the written words. The second condition elaborates on the first condition. Patients were asked to switch between words without blocks, in which they again were asked to name the color of the ink, and words surrounded with blocks, where they had to read the written word out loud. The total time in seconds and the number of errors and self-corrections for both conditions were collected. Finally, a dot counting test, based on the Cortical Vision Screening Test (CORVIST) and the Visual Object and Space Perception Battery (VOSP) [17, 33] was carried out to measure visual perception. Five pictures were shown with a number of dots varying from three to six. Patients were asked to count the number of dots. The picture in the background was the same in all five items and provided a distraction for the patient. The total number of correctly counted items was collected.

### Procedure

The cognitive screener was conducted by means of a tablet and mean duration of the examination was 5–10 min. Data of a measurement was only used if it had been completed for at least 80%. The complete data of an individual patient was only used if at least two measurements out of four had been carried out. The cognitive screening was administrated by a clinical neuropsychologist at four different moments in time; (A) on average 2.5 days before surgery (SD *=* 1.6*)* (preoperative); (B) during surgery, immediately after the tumor resection had been completed and hemostasis and wound closure was being performed; (C) on average 1.6 days after surgery (SD = 0.9) (early postoperative); and (D) weeks after surgery (late postoperative). Measurement D was performed as part of a comprehensive postoperative neuropsychological assessment; for the patients with a high-grade tumors (WHO grade III and IV), this was planned earlier in time (mean = 19.4 weeks, SD = 6.0) than for patients with low-grade tumors (WHO grade II) (mean = 29.6 weeks, SD = 5.5) given their decreased life expectancy.

### Measurement B

Measurement B was less frequently administered than the other measurements; in 26 patients, it was feasible to administer our cognitive screener during the procedure of the awake brain tumor surgery after resection before closure. Fatigue was one of the main reasons measurement B was not performed. Moreover, the focus was on the clinical relevance and the number of tasks administered during the monitoring differed between patients based on location of the tumor and the performance of the patients. Table 2 gives an overview of the neuropsychological functions that were monitored during awake surgery. Therefore, only the best functioning and fittest patients were able to complete the screener during surgery. As a result, performances on group level are biased and cannot be interpreted reliably. Appendix B shows the scores of this measurement.

**Table 2:** Overview of neuropsychological functions monitored during awake brain surgery

Cognitive domain	*n*	%
Language/speech	26	72
Motor skills/sensibility	19	51
Executive functions	18	49
Working memory	10	27
Proprioception	10	27
Visuoperception	5	14
Social cognition	4	11
Clock reading	4	11
Calculation	3	8
Face recognition	2	5
Left right orientation	2	5
Body schema	1	3
Spatial attention	1	3

### Surgical procedure

All participants underwent awake brain surgery under cognitive monitoring by means of an awake-awake-awake procedure under local anesthesia, with microscope view and ultrasound and neuronavigation guidance. Nine patients (24.3%) underwent their procedures in the setting of recurrent tumors. The procedures were performed in a park bench position, allowing the patients to relax and to face the anesthetist and/or the neuropsychologist in a comfortable fashion. The head was fixed in a Mayfield clamp placed under local anesthesia (using a mix of 5mg/ml chirocaine 1:1 v:v and 2% lidocaine with adrenaline 1:200,000 (a total of 29–55cc for the complete procedure) injected at the pin sites of the Mayfield clamp and in a rectangular fashion around the planned skin incision site. After removal of the bone flap using a high-speed Anspach® drill, anesthetics mix-soaked gelatin-foam was applied onto the dura at the level of the meningeal arteries. Patients also received titrated pain sedation and relaxation with remifentanil and propofol, respectively. Tumors were approached using standard routes, and removed using the CUSA at ultra-low power under constant cortico-subcortical stimulations using a Ojeman® cortical stimulator (50Hz, 1ms, 2–4 mAmps, trains of 3 s at the cortical level and continuous stimulations at the subcortical level). None of the patient’s local anesthesia had to be converted to general anesthesia. Areas of stimulation that provoked deficits in the concomitantly performed neuropsychological tests reliably (i.e., 3 times) were considered as functional for that test. In addition, subcortical stimulations that provoked phosphenes, even only once, were considered ass belonging to the optic radiations and respected.

### Statistical analysis

Descriptive and clinical statistics (age, education, gender, and tumor characteristics) were collected. Statistical analyses were performed with Statistical Package for the Social Sciences (SPSS) 26 to compare the cognitive performances. To analyze cognitive function over time, a repeated measures analysis of variance (ANOVA) was performed for normally distributed data. We performed a Friedman ANOVA instead, if data was not normally distributed. To evaluate a significant change, a paired *T* test was conducted. In case of a non-normal distribution, a Wilcoxon related-sample test was carried out. Three measurements were taken into account (A-C-D). Therefore, the significance level for the post hoc was set at alpha .05/3 (*p* < .017).

### Tumor volumetry and measurement of the extent of resection

Tumor volumes were assessed by one of the neurosurgeons (PR) on the immediate preoperative volumetric preoperative MRI images, using, respectively, the FLAIR or gadolinium-enhanced T1modalities and the Brainlab® planning station software for volumetric tumor segmentation. Tumor residuals were obtained in the same fashion using postoperative images obtained within 72 h of the surgery.

## Results

When analyzing the test results with the Friedman Test (A-C-D), a significant effect over time was found for the reaction time in the inhibition task *X*^*2*^ (2, *n =* 24), *p =* .005. The median (*Mdn*) scores in reaction time were the highest at measurement C (*Mdn =* 16.5), followed by A *(Mdn =* 14.5), followed by D *(Mdn = 13.5).* A Wilcoxon signed-rank test indicated a significant difference between C and A (z = −2.85, *p* = .004); Patients needed more time for the inhibition task days after surgery, compared to days before the surgery. Patients showed a trend to improve at measurement D, compared to C (z = −2.38, *p* =.017). The Wilcoxon signed-rank test showed no significant difference between A and D (z = −.07, *p* = .944). The number of errors on the inhibition task did not significantly differ over time *X*^*2*^ (2, *n =* 24), *p* = .174. The median scores did not change over time (*Mdn =* 0). The number of self-corrections were also non-significant *X*^*2*^ (2, *n =* 24), *p = .*529; the median scores were the same (*Mdn =* 0). Other cognitive tasks did not show any significant effects over time. Table 3 summarizes this data.

**Table 3:** Results of neuropsychological tests over time

	Time Inhibition task	Errors Inhibition task	Self-corrections Inhibition task	Time Switching task	Errors Switching task	Self-corrections Switching task	Object naming task	Reading five words	Reading five sentences	Short story errors	Visual perception task	Attention span task	Working memory task
*df, n*	2, 24	2, 24	2, 24	2, 22	2, 22	2, 22	2, 26	2, 26	2 26	2, 25	2, 26	2, 26	2, 26
*p*	.005	.174	.529	.349	.239	.433	.108	.368	.584	.158	.135	.773	.417
*Mdn* A	14.5	0	0	20.5	0	0	15	5	5	0	5	3	3
*Mdn C*	16.5	0	0	19	0	0	15	5	5	1	5	3	2
*Mdn D*	13.5	0	0	19	0	0	15	5	5	0	5	3	2.5

### Extent of resection

A mean extent of resection (EoR) for all cases amounted to a mean of 83.6±21.3 % (range: 1.5–100), with a median of 92.3%. One patient, with a low-grade glioma, presented hand motor function over the entire surface of the tumor, a low-grade astrocytoma, and opted intraoperatively for biopsy rather than resection (intraoperative shared decision-making). In high-grade gliomas, a mean EoR of 88.6±18%, with a median of 96.2%. In low-grade gliomas, these EoR values were, respectively, 77±24.1% and 79.2% or 82±13.7% and 81.6% when excluding the patient who chose intraoperatively for a biopsy. The mean EoR was significantly smaller in surgeries performed for tumor recurrence (71.3%) than in primary surgeries (87.5%, *p* <.05), where 67.9% of the patients underwent a gross total resection.

## Discussion

Our study shows that cognitive functioning remained stable regarding object naming, reading, attention span, working memory, switching, and visuoperception after the surgery. The results are therefore in line with previous retrospective research [22, 30].

Although cognitive functioning overall remains stable, patients in our study were significantly slower on the inhibition task, directly after surgery. This improved in the first months after surgery, returning to baseline. The inhibition task used in this study, based on the CWIT, measures different cognitive functions (e.g., inhibition, attention, processing speed, cognitive flexibility, and working memory) [11]. Surprisingly, we did not find a comparable effect on the switching task, though there are many similarities between those two tasks. At first glance, the switching condition even seems to be more difficult than the inhibition condition. However, Lippa and Davis [20] showed this does not appear to be the case in practice. In their study, participants showed a faster response on the switching task than the inhibition task (absolute rate). This result is possibly due to faster generation of verbal information; in the switching condition there are more items to read in contrast to inhibiting [20]. Moreover, it is possible that a training effect is observed [20] since the inhibition task is always examined before the inhibition/switching task. Based on our data, we cannot draw firm conclusions on what cognitive domain is exactly responsible for the temporary slower performance on the inhibition task. Inhibition seems most likely, but a delayed information processing speed may also play a role. Inhibition is one of the executive functions that is important for self-regulation [16] and everyday life [4]. Furthermore, multiple studies show that information processing speed is an important predictor of quality of life in different clinical populations [2, 29]. With this in mind, advice can be given to the patient in terms of psychoeducation. Patients are already informed about (possible) brain swelling, fatigue, and pain after awake brain surgery. In addition, information about cognitive functioning in the first days after surgery can also be helpful.

One must also ponder the potential deleterious effects of limiting tumor resection due to performing cognitive monitoring in addition to classical language and motor testing. Gross total resection was however achieved in 67.9% of our patients operated for primary tumors, which is at the lower end of the confidence interval of a previously published meta-analysis of gliomas operated with intraoperative monitoring (66–82% of gross total resections). While the EoR in gliomas has been strongly linked to patient survival in retrospective studies [15, 26]. The potential cost of loss of function, especially that of cognitive function and KPS, on survival still needs to be determined [1]. While we do not have enough follow-up on the patients included in this work to answer this important question, we doubt that our surgical policy is oncological detrimental to the patients. We have indeed performed similar testing for years at our center, and our previously published results of patient survival both in low- and high-grade gliomas still compare rather favorably with those of the literature and of similar centers [7, 24]

A strength of this study is the heterogeneous patient group in terms of tumor grade and tumor location. It is therefore well describing the variety of the oncological picture of patients with glioma who are eligible for awake surgery. Furthermore, cognitive functioning was measured both pre- and postoperatively, including days after surgery, in contrast to other available studies [22, 30]. Another strength is the use of a cognitive screener, so that we were able to test patients with brain tumors days after surgery. A comprehensive neuropsychological assessment would be too burdening for a patient at that moment. To our knowledge, this has not been done in previous studies. Although a cognitive screener is not as complete as a comprehensive neuropsychological assessment, cognitive functioning was tested broadly. Literature shows that historically, language, and motor functions are the most tested cognitive domains in awake brain surgery [25]. Other cognitive domains are underexposed but are also important for a good quality of life. For example, good executive functioning is essential for physical and mental health, but also for school and job success [12]. Therefore, during such procedures, it is important not to solely focus on language, but to test cognitive functions broadly [13].

A limitation of the study is that only patients who were able to complete at least two out of four measurements were included in this study. It seems reasonable that these included patients had a relatively better starting position with a larger cognitive reserve/better cognitive resistance than the excluded patients and thereby more favorable preconditions for a stable cognitive recovery course postoperatively. This adds to the uncertainty regarding the representativity of the study group evoked by the small study sample. The above mentioned relatively small sample size precludes an analysis of different other variables that could influence cognitive functioning. For instance, some patients received radiotherapy and chemotherapy after surgery. These kinds of treatments may influence cognitive functioning and may therefore have an effect on measurement D. Another restriction is the fact that we did not include a separate task measuring information processing speed, which could have shed more light on which cognitive domain is responsible for the temporary decline in speed in the inhibition task. This could be included in future studies. Another limitation is that not all cognitive domains were covered in the cognitive screener (e.g., social cognition and episodic memory). Dadario et al. [6] state that these higher-order functions are important and deficits in these cognitive domains can occur after surgery. In this study, we balanced to be concise but also the keep the screener feasible and operable. It is not a comprehensive neuropsychological assessment. Moreover, episodic memory tasks are complicated to administer during surgery [9, 27], because of the time-window of the electric stimulation. We chose to administer a working memory task, as this is conditional to either adequate memory function. However, we do realize that this does not measure the exact same cognitive process. Finally, administration of the screener at moment B (during surgery) was only feasible in a relatively small number of patients due to planning, logistic reasons, and mental and physical condition (e.g., fatigue). Therefore, this measurement was not included in the analyses.

## Conclusion

In conclusion, the results of the current study show that cognitive functioning remains stable in patients that underwent awake brain tumor surgery with cognitive monitoring. We did find that patients needed more time on the inhibition task directly after surgery, but they improved in the following months. However, limited generalizability of these findings should be taken into account. This more detailed timeline of cognitive functioning, in combination with future research, can possibly be contributing in informing patients and caregivers what to expect after awake brain tumor surgery.

## Appendix

See Appendix A and B.

**Appendix A:** Reasons of missing data

Amount of patients	Reason
1	Patient passed away a few weeks after surgery
1	Premorbid psychiatric symptoms
1	Due to very limited neurocognitive capacity surgery was performed under general anesthesia
1	Patient could not participate in the study due to custody
7	< two complete cognitive measurements
1	Patient was incapable of expressive language

**Appendix B:** Descriptive statistic measurement B (during awake brain surgery)

	Time Inhibition task	Errors Inhibition task	Self-corrections Inhibition task	Time Switching task	Errors Switching task	Self-corrections Switching task	Object naming task	Reading five words	Reading five sentences	Short story errors	Visual perception task	Attention span task	Working memory task
*n*	23	23	23	18	18	18	26	26	26	25	26	26	26
*Mdn*	18	0	0	21	0	0	15	5	5	1	5	3	2
25th percentile	12	0	0	16.75	0	0	14	5	4	0	5	2	1.75
75th percentile	23	2	0	28.25	1	1	15	5	5	4	5	3	3

## References

[1] Awad AW, Karsy M, Sanai N, Spetzler R, Zhang Y, Xu Y, Mahan MA (2017). Impact of removed tumor volume and location on patient outcome in glioblastoma. J Neuro-Oncol.

[2] Barker-Collo SL (2006). Quality of life in multiple sclerosis: does information-processing speed have an independent effect?. Arch Clin Neuropsychol.

[3] Brown T, Shah AH, Bregy A, Shah NH, Thambuswamy M, Barbarite E, Fuhrman T, Komotar RJ (2013). Awake craniotomy for brain tumor resection: the rule rather than the exception?. J Neurosurg Anesthesiol.

[4] Chan RC, Shum D, Toulopoulou T, Chen EY (2008). Assessment of executive functions: review of instruments and identification of critical issues. Arch Clin Neuropsychol.

[5] Chang EF, Breshears JD, Raygor KP, Lau D, Molinaro AM, Berger MS (2017). Stereotactic probability and variability of speech arrest and anomia sites during stimulation mapping of the language dominant hemisphere. J Neurosurg.

[6] Dadario NB, Brahimaj B, Yeung J, Sughrue ME (2021). Reducing the Cognitive Footprint of Brain Tumor Surgery. Front Neurol.

[7] De Witt Hamer PC, Ho VKY, Zwinderman AH, Ackermans L, Ardon H, Boomstra S, Bouwknegt W, van den Brink WA, Dirven CM, van der Gaag NA, van der Veer O, Idema AJS, Kloet A, Koopmans J, Ter Laan M, Verstegen MJT, Wagemakers M, Robe PAJT (2019). Quality Registry Neuro Surgery glioblastoma working group from the Dutch Society of Neurosurgery. Between-hospital variation in mortality and survival after glioblastoma surgery in the Dutch Quality Registry for Neuro Surgery. J Neuro-Oncol.

[8] De Witt Hamer PC, Robles SG, Zwinderman AH, Duffau H, Berger MS (2012). Impact of intraoperative stimulation brain mapping on glioma surgery outcome: a meta-analysis. J Clin Oncol.

[9] De Witte E, Satoer D, Colle H, Robert E, Visch-Brink E, Mariën P (2015). Subcortical language and non-language mapping in awake brain surgery: the use of multimodal tests. Acta Neurochir.

[10] De Witte E, Satoer D, Robert E, Colle H, Verheyen S, Visch-Brink E, Mariën P (2015). The Dutch Linguistic Intraoperative Protocol: a valid linguistic approach to awake brain surgery. Brain Lang.

[11] Delis D, Kaplan E, Kramer J (2001). Delis-Kaplan Executive Function System (D-KEFS).

[12] Diamond A (2013). Executive functions. Annu Rev Psychol.

[13] Duffau H (2021). New philosophy, clinical pearls, and methods for intraoperative cognition mapping and monitoring “à la carte” in brain tumor patients. Neurosurgery.

[14] Gerritsen JKW, Zwarthoed RH, Kilgallon JL, Nawabi NL, Jessurun CAC, Versyck G, Pruijn KP, Fisher FL, Larivière E, Solie L, Mekary RA, Satoer DD, Schouten JW, Bos EM, Kloet A, Nandoe Tewarie R, Smith TR, Dirven CMF, De Vleeschouwer S, Broekman MLD, Vincent AJPE (2022). Effect of awake craniotomy in glioblastoma in eloquent areas (GLIOMAP): a propensity score-matched analysis of an international, multicentre, cohort study. Lancet Oncol.

[15] Hervey-Jumper SL, Zhang Y, Phillips JJ, Morshed RA, Young JS, McCoy L, Lafontaine M, Luks T, Ammanuel S, Kakaizada S, Egladyous A, Gogos A, Villanueva-Meyer J, Shai A, Warrier G, Rice T, Crane J, Wrensch M, Wiencke JK et al (2023) Interactive effects of molecular, therapeutic, and patient factors on outcome of diffuse low-grade glioma. J Clin Oncol JCO2102929. 10.1200/JCO.21.02929 10.1200/JCO.21.02929 PMC10082290 36599113

[16] Hofmann W, Schmeichel BJ, Baddeley AD (2012). Executive functions and self-regulation. Trends Cogn Sci.

[17] James M, Plant GT, Warrington EK (2001). CORVIST: cortical vision screeningtest: manual & test materials.

[18] Kim N, Yang J, Lee KS, Shin IS (2021). The effects of preoperative education for patients with cancer: a systematic review and meta-analysis. Cancer Nurs.

[19] Lanenga, I. B (2020). Monitoring cognition before, during and after awake brain tumor surgery; reliability of a cognitive screener (masterthesis). https://studenttheses.uu.nl/handle/20.500.12932/36115

[20] Lippa SM, Davis RN (2010). Inhibition/switching is not necessarily harder than inhibition: an analysis of the D-KEFS color-word interference test. Arch Clin Neuropsychol.

[21] Meng L, Berger MS, Gelb AW (2015). The potential benefits of awake craniotomy for brain tumor resection: an anesthesiologist’s perspective. J Neurosurg Anesthesiol.

[22] Motomura K, Chalise L, Ohka F, Aoki K, Tanahashi K, Hirano M, Nishikawa T, Yamaguchi J, Shimizu H, Wakabayashi T, Natsume A (2019). Neurocognitive and functional outcomes in patients with diffuse frontal lower-grade gliomas undergoing intraoperative awake brain mapping. J Neurosurg.

[23] Prat-Acín R, Galeano-Senabre I, López-Ruiz P, Ayuso-Sacido A, Espert-Tortajada R (2021). Intraoperative brain mapping of language, cognitive functions, and social cognition in awake surgery of low-grade gliomas located in the right non-dominant hemisphere. Clin Neurol Neurosurg.

[24] Robe PA, Rados M, Spliet WG, Hoff RG, Gosselaar P, Broekman MLD, van Zandvoort MJ, Seute T, Snijders TJ (2022). Early surgery prolongs professional activity in IDH mutant low-grade glioma patients: a policy change analysis. Front Oncol.

[25] Ruis C (2018). Monitoring cognition during awake brain surgery in adults: a systematic review. J Clin Exp Neuropsychol.

[26] Sanai N, Polley MY, McDermott MW, Parsa AT, Berger MS (2011). An extent of resection threshold for newly diagnosed glioblastomas. J Neurosurg.

[27] Skrap M, Marin D, Ius T, Fabbro F, Tomasino B (2016). Brain mapping: a novel intraoperative neuropsychological approach. J Neurosurg.

[28] Snodgrass JG, Vanderwart M (1980). A standardized set of 260 pictures: norms for name agreement, image agreement, familiarity, and visual complexity. J Exp Psychol Hum Learn.

[29] Ueoka Y, Tomotake M, Tanaka T, Kaneda Y, Taniguchi K, Nakataki M, Numata S, Tayoshi S, Yamauchi K, Sumitani S, Ohmori T, Ueno S, Ohmori T (2011). Quality of life and cognitive dysfunction in people with schizophrenia. Prog Neuro-Psychopharmacol Biol Psychiatry.

[30] Van Kessel E, Snijders TJ, Baumfalk AE, Ruis C, van Baarsen KM, Broekman ML, van Zandvoort MJE, Robe PA (2020). Neurocognitive changes after awake surgery in glioma patients: a retrospective cohort study. J Neuro-Oncol.

[31] Van Zandvoort M, Ruis C, Hendriks M (2016). Wakkere hersenoperaties: de klinisch neuropsychologisch aspecten. Neuropraxis.

[32] Verhage F (1964). Intelligentie en Leeftijd Onderzoek bij Nederlanders Van Twaalf tot Zevenenzeventig Jaar [Intelligence and age research with Dutch people aged twelve to seventyseven years].

[33] Warrington EK, James M (1991). The visual object and space perception battery.

[34] Wechsler D (2008). Wechsler adult intelligence scale–fourth edition (WAIS–IV).

[35] Whittle IR, Midgley S, Georges H, Pringle AM, Taylor R (2005). Patient perceptions of “awake” brain tumour surgery. Acta Neurochir.

